# Comparative In Vitro Assessment of a Range of Commercial Feed Additives with Multiple Mycotoxin Binding Claims

**DOI:** 10.3390/toxins11110659

**Published:** 2019-11-12

**Authors:** Oluwatobi Kolawole, Julie Meneely, Brett Greer, Olivier Chevallier, David S. Jones, Lisa Connolly, Christopher Elliott

**Affiliations:** 1 Institute for Global Food Security, Queens University Belfast, Belfast BT9 SDL, UK; okolawole01@qub.ac.uk (O.K.); j.p.meneely@qub.ac.uk (J.M.); brett.greer@qub.ac.uk (B.G.); o.chevallier@qub.ac.uk (O.C.); l.connolly@qub.ac.uk (L.C.); 2 School of Pharmacy, Queens University Belfast, Belfast BT9 7BL, UK; D.Jones@qub.ac.uk

**Keywords:** mycotoxins, animal feed, mycotoxin binders, feed safety

## Abstract

Contamination of animal feed with multiple mycotoxins is an ongoing and growing issue, as over 60% of cereal crops worldwide have been shown to be contaminated with mycotoxins. The present study was carried out to assess the efficacy of commercial feed additives sold with multi-mycotoxin binding claims. Ten feed additives were obtained and categorised into three groups based on their main composition. Their capacity to simultaneously adsorb deoxynivalenol (DON), zearalenone (ZEN), fumonisin B1 (FB1), ochratoxin A (OTA), aflatoxin B1 (AFB1) and T-2 toxin was assessed and compared using an in vitro model designed to simulate the gastrointestinal tract of a monogastric animal. Results showed that only one product (a modified yeast cell wall) effectively adsorbed more than 50% of DON, ZEN, FB1, OTA, T-2 and AFB1, in the following order: AFB1 > ZEN > T-2 > DON > OTA > FB1. The remaining products were able to moderately bind AFB1 (44–58%) but had less, or in some cases, no effect on ZEN, FB1, OTA and T-2 binding (<35%). It is important for companies producing mycotoxin binders that their products undergo rigorous trials under the conditions which best mimic the environment that they must be active in. Claims on the binding efficiency should only be made when such data has been generated.

## 1. Introduction

Mycotoxins are toxic, low-molecular weight compounds produced as secondary metabolites by several fungi species belonging mainly to *Aspergillus*, *Fusarium*, *Penicillum, Alternaria* and *Clavicep* genera [1]. Under favourable environmental conditions such as moisture and temperature, these fungi can invade crops and proliferate (during growth, transportation and storage) to produce mycotoxins [2]. Other factors including climate change, poor harvesting practices, improper drying, handling and packaging may also predispose crops to fungal invasion and subsequent mycotoxin production [3]. Mycotoxins appear in the food and feed chain because forages and cereals, which are most susceptible crops to these fungi, are utilised as the main components of animal feed. Among the more than 400 mycotoxins currently identified, aflatoxin B1 (AFB1), deoxynivalenol (DON), zearalenone (ZEN), ochratoxin A (OTA), fumonisins B1 (FB1) and trichothecenes T-2/HT-2 toxin are considered the most economically significant mycotoxins in terms of their prevalence and their negative effects on human and animal health and performance [4,5]. In addition to the well characterised fungal mycotoxins, biological metabolism or modification of mycotoxins mainly by plants can lead to conjugated forms of mycotoxins widely known as masked mycotoxins. These mycotoxin derivatives are often not detected by analytical techniques, and several studies have shown them to be a potential threat to consumers, as they can be converted to their parent forms in the gastrointestinal tract (GIT) after ingestion [1].

DON, T-2, ZEN and FB1 are produced by *Fusarium* species including *Fusarium graminearum*, *Fusarium sporotrichioides, Fusarium verticilloides and Fusarium poae* [4]. DON and T-2 are potent DNA protein synthesis inhibitors and cause digestive disorders, oral lesions, immunologic effects and hematological disorder [6,7]. ZEN is estrogenic and impair reproductive performance [8]. FB1 is associated with liver necrosis, diarrhoea, intestinal disorder, nephritis and oedema [9]. OTA is produced by species of *Aspergillus* and *Penicillium* (mainly *Aspergillus ochraceus* and *Penicillium verrucosum)*. OTA exerts several toxic effects including nephrotoxicity, hepatogenicity and genotoxicity; it can also affect carbohydrate metabolism and blood coagulation [10]. AFB1 is produced by *Aspergillus* species (*Aspergillus flavus* and *Aspergillus parasiticus*). AFB1 health effects include teratogenicity, mutagenicity and carcinogenicity, with the liver being the primarily affected organ [11]. In farm animals, ingestion of a diet contaminated with more than one mycotoxin may cause more complex additive, antagonistic or synergistic effect on health and performance [12]. The severity of symptoms depends on a number of factors including the type of mycotoxin and level present, animal species, gender, diet, age and duration of exposure. In addition, diagnosis is difficult as mycotoxicosis produces a very wide variety of clinical signs [13].

Mycotoxin occurrence in cereal crops sits at 25% in terms of breaches of European Union (EU) Codex limits and occurrence above the detectable levels ranges from to 60–80% depending on the crop type [4]. To minimise the negative effects of these mycotoxins in farm animals, several methods have been developed to reduce the occurrence of mycotoxins in animal feeds, these include physical (thermal and irradiation) [14,15]; chemical (ozonation and ammoniation) [16,17] and biological (microorganisms and enzymes) [18,19]. However, inclusion of binders or adsorbents to feed as a form of additive, appears to be the most prevalent strategy widely practiced by farmers and the feed industry, due to its economic feasibility [20]. The additives are added to the diet of animals to reduce the absorption of mycotoxins from the GIT and their distribution to blood and target organs [21]. The additives used for this purpose have been divided into two groups: binders and modifiers. Mycotoxin binders aim to prevent the absorption of the mycotoxins from the intestinal tract of the animal by adsorbing the toxins to their surface to form a mycotoxin-binder complexes, which are then excreted in animal faeces. Mycotoxin binders are mainly classified as: organic (yeast cell wall and glucommanan) and inorganic (clay minerals such as aluminosilicate, bentonite and zeolite) [22]. Mycotoxin modifiers are of biological origin (bacteria, fungi, enzymes and plants); they alter the chemical structure of mycotoxins (biotransformation) to produce metabolites that are less toxic than the parent mycotoxins or non-toxic [23]. Throughout this paper, terms including adsorption, binding and sequestering, will be used to describe the reduction or removal of mycotoxins by feed additives.

In the EU, there is a provision under European Commission (EC) regulation (EC 1831/2003), for the inclusion of a technical additive—”a substance that can suppress or reduce the absorption, promote the excretion of mycotoxins or modify their mode of action”- to animal feed. To register a feed additive in the EU, an application must be submitted to the EC, a technical dossier to European Food Safety Authority (EFSA) and three reference samples of the feed additive must be sent to European Union Reference Laboratory, who evaluates the safety and efficacy of the samples before they can be authorized for use in the EU. However, in many other parts of the world, there are no regulations regarding the use of feed additives or substances that can counteract the toxic effects of mycotoxins in farm animals [20].

Several of these products are registered as digestibility enhancers, antioxidants and generic or catalogue names such as montmorillonite, bentonite and hydrated sodium calcium aluminosilicate (HSCAS), with mycotoxin binding claims [24,25]. Many researchers have investigated mycotoxin sequestering potentials of some of these products, which are commercially available worldwide. However, most of the studies are focused on only one or two mycotoxins, particularly AFB1 [26,27,28]. As mycotoxins are often co-occurring in animal feed [29,30], the current study aims to evaluate and compare the efficacy of ten commercial feed additives with multi-mycotoxin binding claims on DON, T-2, ZEN, OTA, FB1 and AFB1 using an in vitro model simulated to mimic the gastro-intestinal tract (GIT) of a monogastric animal. Results showed that only one of the products (a modified yeast cell wall) effectively adsorbed more than 50% of DON, ZEN, FB1, OTA, T-2 and AFB1, in the following order: AFB1 > ZEN > T-2 > DON > OTA > FB1. The remaining products were able to moderately bind AFB1 (44–58%) but had less, or in some cases, no effect on ZEN, FB1, OTA and T-2 binding (<35%).

## 2. Results and Discussion

Contamination of different agricultural commodities with multi-mycotoxins, as well as adverse health effects and reduction in animal performance, due to mycotoxicosis are still prevalent, despite the prevention strategies currently employed [31,32,33]. Additives are added to the diets of livestock animals to bind mycotoxins and reduce their bioavailability in GIT and distribution to blood and target organs. The most prevalent adsorbing agents are polymers, yeast cell wall, cholestyramine and clay minerals [21,22]. In vitro analysis of mycotoxin adsorption is a very useful tool for rapid screening and identification of agents that may have mycotoxin sequestering potentials [22]. Several researchers have investigated different mycotoxin binders, however, most of the studies have focused on a single mycotoxin and carried out using buffer solutions mostly at pH 3 and 7, to simulate physiological pH in stomach and intestine, respectively. This does not truly reflect the conditions in a farm animal GIT as other factors including temperature, digestive enzymes, feed, bile salts and nutrients may interfere with the adsorption (ion-exchange) process [21].

In the current study, ten commercial feed additives with adsorption, inactivation or detoxification claims on DON, ZEN, FB1, OTA, T-2 and AFB1 were obtained and categorised into three groups based on their composition. Their capacity to simultaneously bind or adsorb DON, ZEN, FB1, OTA, T-2 and AFB1, which often co-occur in complete feed or feed ingredients such as maize, wheat and barley was assessed and compared. The ratio of additive:binder used in this study is based on the maximum permitted/guidance levels for mycotoxins in European pig feed [34] and the conventional binder inclusion level of 2 g/kg feed [35]. In order to assess the mycotoxin binding capacity of the adsorbents, an in vitro system with buffer solutions at pH 3 and 7—to simulate stomach and intestine respectively, was used to study mycotoxin adsorption/desorption. Furthermore, a robust in vitro model relative to the GIT of a monogastric animal in terms of compartment, enzymes, feed, gastric fluids, temperature, pH and transit time was designed, to investigate the adsorption efficacy of the feed additives. The percentage adsorption of DON, ZEN, FB1, OTA, T-2 and AFB1 by various feed additives in buffer solutions as well as in vitro GIT model are presented in Table 1 and Table 2, respectively.

### 2.1. Inorganic Additives

Aluminosilicate constitute the most abundant group of rock-forming minerals [36]. The basic structural unit of silicate clay minerals consists of the combination of aluminium octahedral and silica tetrahedral sheets, both with hydroxyl and oxygen groups [37]. Most studies (both in vivo and in vitro) on mycotoxin binders using clay minerals have focused on aluminosilicates such as bentonite, montmorillonite, zeolite and hydrated sodium calcium aluminosilicates (HSCAS). They possess high cation exchange capacity, pore volume and large surface area, which enable them to adsorb low-molecular weight compounds such as mycotoxins to their surfaces, edges and interlayer spaces [27]. Four commercial clay-based products (1, 2, 3 and 4) were investigated for their multi-mycotoxin binding potentials. Results obtained for in vitro buffer solutions showed that all the 4 products bound DON, ZEN, FB1, OTA, T-2 and AFB1 at adsorption rates of 29–58%, 27–42%, 29–47%, 5–40%, 9–38%, 51–68% respectively (Table 1). Product 1 and 3 had a significant adsorption on AFB1 (68% and 61%), DON (53% and 49%) and ZEN (42% and 46%) respectively, compared to product 2 and 4 (*p* < 0.05). There was no significant difference (*p* > 0.05) in the adsorption of DON, ZEN, FB1, OTA, T-2 and AFB1 by product 2 and 4, as they sequestered <34% of DON, ZEN and FB1; <13% of OTA and T-2, and approximately 50% of AFB1. Within this category, AFB1 was the most adsorbed mycotoxin followed by DON, ZEN, FB1, OTA and T-2 at pH 3 and 7. Several studies on adsorption of mycotoxins using buffer solutions at different pH (mostly 3, 5 and 7) have shown that AFB1 is highly adsorbed by clay minerals at acidic and alkaline pH with little to no adsorption of other mycotoxins [26,38,39]. A recent study on the efficacy of commercial clay minerals to sequester 0.1 µg/mL of AFB1, DON and ZEN showed that 1% of a commercial smectite and an aluminosilicate clays significantly adsorbed AFB1 (95–100%) and ZEN (56–82%) in acidic pH, with no significant effect on DON (<10%) [40]. Similarly, 50 mg of a commercial bentonite adsorbed 99% of 10 ng/mL AFB1 and 1% of 250 ng/mL DON in buffer solution (pH 5) [41]. The difference in the ability of clay minerals to sequester mycotoxin has been attributed to their origin and physiochemical properties such as cation exchange capacity, pore volume and expandability [21,22].

For the in vitro GIT experiment, 22–100% reduction in the efficacy of all the four clay-based products to adsorb multi-mycotoxins was observed (Table 2). Adsorption capacities of product 2 and 4 on DON, ZEN and FB1 were the most severely affected. Their adsorption rates were reduced to less than 23%, 18% and 16% for DON, ZEN and FB1, respectively, with no observed adsorption of OTA and T-2. However, both products (2 and 4) were still able to significantly bind >42% of AFB1 (*p* < 0.05) (Table 2). Product 1 performed better than other products within this group, with a simultaneous adsorption rate of 46%, 33%, 29%, 25%, 26% and 51% for DON, ZEN, FB1, OTA, T-2 and AFB1 respectively, followed by product 3: DON (37%), ZEN (29%), FB1 (22%), OTA (18%), T-2 (15%) and AFB1 (53%). A similar study carried out by Vekiru et al. [42], showed that the sequestering potential of HSCAS, activated charcoal and bentonites against AFB1 strongly decreased in the presence of swine gastric juice. The percentage adsorption dropped from 98% to 72% for HSCAS, 88% to 35% for activated charcoal and by more than 15% for bentonites [42]. Also, the capacity of 1 mg of smectite to reduce 8 µg/mL aflatoxin was reduced from 0.5 mol/kg in distilled water to 0.2 mol/kg in simulated gastric fluid [43].

Although, the adsorption capacity of product 1 and 3 were reduced in the GIT model, they still significantly adsorbed DON, ZEN, FB1, OTA, T-2 and AFB1 (*p* < 0.05) when compared with product 2 and 4. These products (1 and 3) are chemically modified clay minerals. Modified clay minerals have been shown to possess high mycotoxin-sequestering ability compare to natural clay minerals (product 2 and 4). Modified adsorbents are prepared by alteration of surface properties such as cation exchange capacity using acids, alkalis, organic compounds and heat, that consequently increase their contaminant removal capacity and efficacy [44]. Nevertheless, their safety and interaction with nutrients and veterinary substances remain a concern [45].

### 2.2. Organic Additives

The three products (5, 6 and 7) within this category of feed additives, were able to bind DON, ZEN, FB1, OTA, T-2 and AFB1 in the range of 36–56%, 28–69%, 19–55%, 35–60%, 10–56% and 55–65%, respectively, mostly at pH 3 (*p* < 0.05) (Table 1). Only product 6 was able to adsorb more than 50% of each toxin simultaneously: DON (55%), ZEN (56%), FB1 (55%), OTA (60%), T-2 (56%) and AFB1 (65%). Product 5 adsorbed DON (56%) and AFB1 (51%), with moderate binding on ZEN, FB1, OTA and T-2 (<45%). Product 7 also adsorbed AFB1 (55%), but its multi-mycotoxin adsorption capacity on DON (36%), ZEN (28%), FB1 (19%), OTA (35%), T-2 (10%) was significantly lower when compared to product 5 and 6 (*p* < 0.05). In terms of percentage adsorption under in vitro GIT model, again, all the products binding capacities were reduced, but to a much lesser extent compared with products under inorganic additives (Table 2). Only product 6 had a significant binding (*p* < 0.05) on ZEN (56%), FB1 (55%), OTA (60%), T-2 (56%) and AFB1 (63%). However, no significant difference was observed in the adsorption of DON, FB1 and AFB1 by products 5 and 6 (*p* > 0.05). Interestingly, the percentage adsorption of ZEN by product 6 in buffer solutions (56%) was similar to that which was found in the in vitro GIT model, which indicates a good additive with high mycotoxin specificity. Similar results were obtained by Joannis-Casssan et al. [46]. This group tested the binding efficacy of a commercial yeast cell wall obtained from baker’s yeast on OTA, AFB1 and ZEN and found it to effectively adsorb up to 62%, 29% and 68%, respectively, in a dose-dependent manner [46]. Also, a commercial inactivated yeast-based product sandwiched with glutathione bound 45% of AFB1 and more than 50% of OTA and ZEN [47].

Organic additives such as yeast cell wall and glucommanan have been shown to have a high binding activity across a wide spectrum of mycotoxins compare to inorganic minerals [48]. The cell wall of the yeast *Saccharomyces cerevisiae* is composed of lipids, protein and polysaccharide fraction, with glucans and mannans being the two main constituents of the latter fraction [47]. Glucomannan is a water-soluble polysaccharide composed of hemicellulose, it is present in the cell wall of some plant species. Several authors have suggested that the cell wall components of these substances could be responsible for the adsorption of mycotoxins through non-covalent, hydrogen bonds, ionic or hydrophobic interactions [49,50,51]. Rignot et al. [50] showed that β-D-glucans are the yeast component largely responsible for the complexation of mycotoxins, and that the reticular organization of β-D-glucans and the distribution between β-(1,6)-D-glucans and β-(1,3)-D-glucans plays a vital role in mycotoxin adsorption [50]. Furthermore, Van der Waals forces and weak hydrogen bonding maybe involved in the adsorption of mycotoxins by β-D-glucans [49]. The efficacy and type of mycotoxins a yeast cell wall product can adsorb is dependent on the origin of yeast, strain, pH, binding sites or accessible surface area, growth condition and percentage of cell wall components (mannoproteins, chitins, lipids and β-glucan) [51]. Glucomannan is commonly used as a dietary fibre, however, there are very limited studies published regarding the types of mycotoxins adsorbed and mechanisms of adsorption.

### 2.3. Mixture of Additives

Due to the affinity of most technical additives towards a single mycotoxin, a mixture of additives has been developed and used recently, to counteract adverse health effects of multiple mycotoxins in farm animals, the most prevalent one being mixture of clay minerals and yeast cell wall [26]. Three commercial products with mixed additives (product 8—mixed silicates and yeast cell wall; product 9—yeast cell wall and enzyme; product 10—natural clay minerals and algae) were assessed for their multi-mycotoxin binding capacity in the present study. Results of in vitro buffer solution testing showed that the three products adsorbed DON (32–72%), ZEN (22–55%), FB1 (19–56%) OTA (15–49%), T-2 (38–55%) and AFB1 (32–63%), mostly at pH 3 (*p* < 0.05) (Table 1). Product 9 significantly sequestered DON (72%), ZEN (55%) and T-2 (55%), however, compared to product 8, no significant adsorption was observed (*p* > 0.05) for FB1 (42%) and AFB1 (63%). Product 10 had a poor binding capacity on DON, ZEN, FB1, OTA and T-2 with an adsorption rate of 32%, 22%, 19%, 15% and 38%, respectively. Under in vitro GIT conditions, product 10 lost its binding potential as mycotoxins adsorption rates were reduced to 22%, 8%, 0%, 0% and 24% for DON, ZEN, FB1, OTA and T-2, respectively. Similar results were obtained for product 8, with a reduction from 52–41%, 46–35%, 39–23%, 20–9%, 43–28% and 61–48% for DON, ZEN, FB1, OTA, T-2 and AFB1, respectively. Product 9 significantly adsorbed DON (61%), ZEN (55%), OTA (33%), T-2 (36%) and AFB1 (58%) compared to product 8 and 10 (*p* < 0.05) (Table 2). Results obtained for products within this category suggest that mere mixing of additives does not guarantee multi-mycotoxin binding as the mixture may either lead to synergistic or antagonistic effect. When mixing additives, it is important to investigate the efficacy of individual agent and their mixtures to identify the mycotoxins they can adsorb effectively.

Generally, all the commercial binder or feed additive products assessed for their capacity to bind multi-mycotoxin in the current study adsorbed DON, ZEN, FB1, OTA, T-2 and AFB1 simultaneously at different rates under both acidic and alkaline pH. However, percentage adsorption at pH 3 was more significant when compare with adsorption at pH 7 (*p* < 0.05), this indicates that products investigated can form a stable mycotoxin-binder complex at pH 3 and to some extent at pH 7. Under in vitro GIT model, adsorption efficacies of all the products were reduced (except product 6, for ZEN) possibly due to interaction of binder products with other components of GIT such as pepsin, HCl and feed [43,52,53]. For instance, Barrientos et al. [43] showed that the adsorption of a globular protein (pepsin) by a smectite clay significantly reduced the adsorption rate of AFB1 in simulated acidic gastrointestinal fluid [43]. Also, a corn protein interfered with AFB1 adsorption to a smectite clay in corn fermentation solution [53].

Regarding the performances of investigated feed additives under the in vitro GIT model, product 1—a modified aluminosilicate had a good multi-mycotoxin binding capacity on DON (46%), ZEN (33%), T-2 (29%), FB1 (25%), OTA (25%) and AFB1 (51%) when compared with other products in this category (*p* < 0.05). Within category of organic additives, a modified yeast extract (product 6) had a broad significant mycotoxin adsorption spectrum, with adsorption rate of 55%, 56%, 51%, 53%, 56% and 65% for DON, ZEN, FB1, OTA, T-2 and AFB1, respectively. Within category of mixture of additives, product 9 (a mixed yeast cell wall and enzymes) adsorbed 61%, 55%, 28%, 33%, 36% and 58% of DON, ZEN, FB1, OTA, T-2 and AFB1, respectively (*p* < 0.05). Overall, in terms of multi-mycotoxin binding efficiency, only product 6 performed well, as it was able to simultaneously sequester more than 50% of mycotoxins in the following order: AFB1 > ZEN > T-2 > DON > OTA > FB1; followed by product 9 (yeast cell wall and enzyme) and product 5 (glucomannan).

Mycotoxin binders adsorb mycotoxin at the surface, to form a mycotoxin-binder complex, the bound mycotoxins are then excreted along with the binder in animal faeces. The adsorption capacity and stability of the complex through the GIT is influenced by physiochemical properties of the binder including polarity, size of the pores and accessible surface area as well as physicochemical properties of mycotoxins including polarity, solubility, size and charge [38]. AFB1 is relatively hydrophilic with aromatic planar molecules, therefore it is easily bound by most binders, particularly clay minerals, under both acidic and alkaline conditions, by formation of a coordination bonds with the beta-carbonyl system [26]. However, other mycotoxins—ZEN, OTA, FB1, T-2 and DON range from being moderately hydrophilic to high hydrophobic compounds, therefore being very difficult to adsorb [26]. However, emerging nanocomposites [54], modified organic and inorganic adsorbents [55] are being used to sequester these mycotoxins.

## 3. Conclusions

In light of the high co-occurrence of fungi and mycotoxins in agricultural commodities, exacerbated by climate change, products with wide spectrum mycotoxin adsorption or detoxification are in great demand from farmers and animal feed producers, to minimise the economic losses caused by mycotoxicosis. In the current study, an in vitro GIT model was designed to assess and compare the efficacy of ten commercially available binder products with multiple mycotoxin claims on DON, ZEN, FB1, OTA, T-2 and AFB1. Results showed that most of the products were able to significantly bind DON, ZEN, FB1, OTA, T-2 and AFB1 in both alkaline and acidic buffer solutions. However, under the in vitro model simulating the conditions in the GIT of monogastric animals such as chicken and pig, the efficacy of all the products were significantly reduced and only one of the products tested (6—a modified yeast cell wall) was still able to simultaneously adsorb more than 50% of DON, ZEN, FB1, OTA, T-2 and AFB1, in the following order AFB1 > ZEN > T-2 > DON > OTA > FB1. The remaining products were able to moderately bind AFB1 (44–58%) but had less than 35% or in some cases no binding effect on ZEN, FB1, OTA and T-2 binding. A robust method that mimics the GIT condition of a farm animal must be used to study the efficiency of a potential mycotoxin binder, not the conventional use of buffers at different pH. Furthermore, producers of feed additives with mycotoxin binding claims should ensure appropriate and detailed labelling of their products such as the composition, physicochemical properties, mode of action, dosage and importantly the specific mycotoxin(s) their product can bind, adsorb or detoxify, to ensure farmers and animal nutrition companies are not misled.

## 4. Materials and Methods

### 4.1. Chemicals and Reagents

Hydrochloric acid (HCl), citric acid, monobasic sodium phosphate (NaH_2_PO_4_), pancreatin, pepsin, polytetrafluoroethylene (PTFE) filter, formic acid, bile salt, sodium chloride (NaCl), sodium bicarbonate (NaHCO_3_), LC-MS grade methanol and acetonitrile were supplied by Sigma-Aldrich (Gillingham, UK). Mycotoxins—AFB1, ZEN, FB1, OTA, T-2 and DON—crystalline solids were obtained from Romer Labs GmbH (Tulln, Austria). Ultra-pure water was obtained from a Milli-Q Gradient A10 water purification device (Millipore, Molsheim, France). All chemicals used were of analytical grade unless otherwise stated.

### 4.2. Feed Additives

Ten commercially available products claiming multiple mycotoxin adsorption or binding on DON, T-2, ZEN, AFB1 and FB1 were obtained and categorised into three groups (inorganic, organic and mixture of additives) based on their main functional composition. The products were coded with numbers to preserve the confidentiality of the source. Products 1, 3 and 4 were purchased online, while products 2, 5, 6, 7, 8, 9 and 10 were obtained directly from the companies. Product details including mode of action and main composition (as stated on the product labels and manufacturers’ websites) are listed in Table 3.

### 4.3. Multi-Mycotoxins Adsorption Experiment

#### 4.3.1. Buffer Solution

Mycotoxin stock solutions (1 mg/mL) of AFB1, DON, ZEN, T-2 and OTA were prepared by dissolving pure solid standards in methanol and FB1 in acetonitrile/water (50:50, *v*/*v*). A mixed-mycotoxin working solution was prepared in 10 mL acetonitrile and stored at −20 °C until use. To evaluate adsorption efficacy of the binding products and stability (adsorption/desorption) of mycotoxin-binders complex in both acidic and alkaline conditions, adsorption capacity of each product was studied at pH 3 and 7 to simulate physiologic pH in the stomach and intestine of monogastric animal respectively. The buffer solutions (pH 3 and 7) were prepared by using 0.1 M citrate and 0.2 M phosphate buffers. Each product (20 mg) was weighed into a 30 mL flask containing 10 mL of buffer solution; 20 µL of multi-mycotoxin working standard solution was added to reach a final concentration of 20 ng/mL, 50 ng/mL, 900 ng/mL, 5000 ng/mL, 100 ng/mL and 250 ng/mL for AFB1, OTA, DON, FB1, ZEN and T-2 respectively, this was performed for each pH in triplicate. A blank control was prepared using only multi-mycotoxin working solution in buffers without any mycotoxin binder. The flasks were shaken and incubated for 3 h at 37 °C in an incubator shaker. Thereafter, samples were centrifuged (30 min, 1000× *g*, 25 °C), and 1 mL of supernatant was mixed with an equal volume of acetonitrile and evaporated to dryness under gentle nitrogen stream (40 °C). The residue was reconstituted in 1 mL of methanol, filtered through 0.2 µm PTFE filter and transferred to a glass vial for LC-MS/MS analyses.

#### 4.3.2. In Vitro Gastrointestinal Model

An artificially contaminated feed was made by spiking 1 g of finely ground feed material with 200 µL of multi-mycotoxin stock solution to reach approximately the following mycotoxin concentrations, based on EU permitted/regulated limit for mycotoxins in pig feed: AFB1 (21.2 µg/kg), OTA (48.9 µg/kg), DON (997.2 µg/kg), FB1 (5582.3 µg/kg), T-2 (243.1 µg/kg) and ZEN (152.8 µg/kg). The spiked material was incubated overnight in the dark at 40 °C to evaporate to dryness. To check the homogeneity of the batch, three samples taken randomly were extracted and analysed for multi-mycotoxins using a previously validated QuEChERS-based LC-MS/MS method [56]. To assess the efficacy of commercial feed additives to adsorb multiple mycotoxins, an in vitro model was designed to simulate the GIT conditions of monogastric animal using an automated dissolution USP Apparatus 2 (Vankel VK 7010, Erweka, Germany) with an auto-controlled multi-channel peristaltic pump (Vankel VK 810, England). Temperature of 40 °C and rotation speed of 100 rpm were used throughout the experiment. The first GIT compartment simulated was the crop/oesophagus, 1.0 g of multi-mycotoxin contaminated feed and 20 mg of each feed additive were mixed with 40 mM of acetic acid, 0.2 M Na_2_ HPO_4_ and 5 M NaCl buffer. Each tube was mixed to reach a pH value of 4.5–5.3 and incubated for 60 min. Subsequent stomach/proventriculus simulation was performed by addition of 0.23 M HCl, 0.034 M NaCl and 5000 U of purified pepsin derived from pig stomach mucosa, to reach a pH between 1.9 and 3.7, tubes were further incubated for 90 min. The final GIT compartment simulated was the intestine; here, 0.05 M NaHCO_3_, pancreatin (0.5 mg/mL) and 0.4% bile salt were added to the tubes, the pH was increased and ranged between 5.3 and 7.5. All samples were incubated further for 120 min. The total incubation time for the in vitro digestion was 4 h and 30 min. Blank controls were prepared without the addition of any feed additive, and all experiments were performed in quintuplicate. After incubation, 1 mL of sample was withdrawn and mixed with 1 mL of 0.01% formic acid in acetonitrile, followed by a rigorous vortex and centrifugation at 10,000× *g* for 30 min. Subsequently, 1 mL of supernatant was dried under gentle nitrogen stream (35 °C) and residue was re-dissolved in 0.5 mL of methanol, filtered through 0.2 µm PTFE filter and transferred to a glass vial for LC-MS/MS analysis.

### 4.4. LC-MS/MS Analysis of Mycotoxins

AFB1, DON, ZEN, T-2, FB1 and OTA were analysed using an Acquity UPLC I-Class system coupled to a Xevo TQ-S triple quadrupole mass spectrometer (MS) (both from Waters, Milford, USA), which allowed the simultaneous determination of the toxins. Data acquisition and instrument control were performed by Masslynx software (Waters, Milford, MA). For the UPLC, the column used was a Cortecs C18 100 mm × 2.1 mm i.d., 1.6 µm (Waters, Milford, USA) and mobile phases consisted of A —water and B—methanol: acetonitrile (1:1, *v*/*v*). Both contained 0.1% formic acid and 1mM ammonium formate. Chromatographic separation was achieved through a gradient elution program as follows: 0–2 min, 99% A/1% B; 2–3 min 30% A/70% B, 3–5.5 min, 1% A/99% B; 5.5–6.5 min, 99%A/1% B; 6.5–7 min, 99% A/1% B. Column temperature was maintained at 40 °C, and the flow rate and injection volume were set at 0.4 mL/min and 1 µL respectively. ESI-MS/MS was performed in a multiple reaction monitoring mode (MRM) at positive polarity. The *m*/*z* transitions for quantification were 313.2 > 241.2, 297.3 > 249.5, 319.3 > 282.9, 466.5 > 245.1, 721.83 > 335.1, 403.8 > 238.8 for AFB1, DON, ZEN, T-2, FB1 and OTA respectively. The ion source parameters were as follows: capillary voltage, 1 kV; desolvation temperature, 600 °C; source temperature, 150 °C; cone gas flow, 50 L/h; desolvation gas flow, 1000 L/h. Collision energy and cone voltage were optimised by an infusion of each compound using the IntelliStart function.

### 4.5. Method Performance

**Figure 1:**
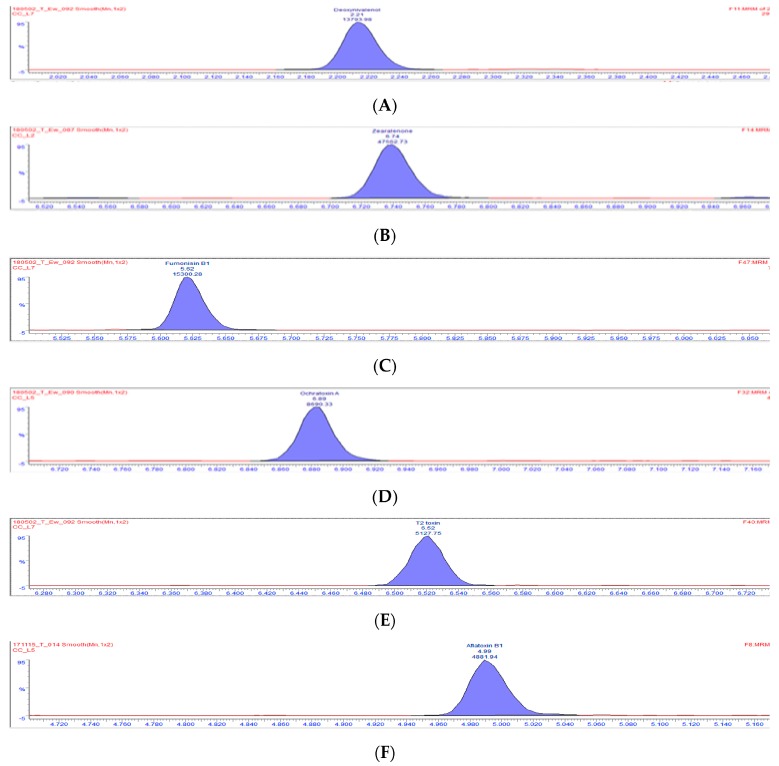
UPLC-MS/MS chromatograms of spiked feed sample (**A**) at 10 µg/kg for deoxynivalenol (DON), (**B**) 5 µg/kg for zearalenone (ZEN), (**C**) 10 µg/kg for fumonisin (FB1), (**D**) 2 µg/kg for ochratoxin A (OTA), (**E**) 5 µg/kg for T-2 toxin, (**F**) 0.5 µg/kg for aflatoxin B1 (AFB1).

For quantification, a seven-point calibration curve was prepared for each mycotoxin in the following concentration range: 0.5−50 ng/mL for AFB1, 10−5000 ng/mL for FB1, 5–500 ng/mL for ZEN, T-2 and OTA, and 10−2000 ng/mL for DON. To evaluate the effects of the matrix on MS quantification, matrix-induced suppression/enhancement (SSE) was determined by comparing the response of matrix spiked with seven different concentrations of each mycotoxin to a neat solvent standard at the same concentrations. The experiment was performed for buffer solutions (pH 3, pH 7) and gastrointestinal fluid (GF) in triplicate at three different times. SSE was calculated as the ratio of calibration curve slope for matrix-matched standards and neat solvent standards multiplied by 100. Limits of detection and limits of quantification (LOQ) of each mycotoxin were calculated at a signal-to-noise ratio of 3:1 and 10:1, respectively, based on a matrix-matched calibration in buffer solutions and GF. The coefficients of determination (R^2^) for selected mycotoxins in the three matrices (pH 3, pH 7 and GF) ranged from 0.9901 to 0.9995. The retention times of the analyte in the sample extract were checked to correspond to that of the calibration standards and was within a tolerance of ± 0.1 min. Also, the ion ratios were within 25% of that obtained from the calibration standard for all analytes. SSE and LOQs values obtained for mycotoxins in the three matrices are reported in Table 4. Figure 1 shows chromatograms obtained for the six mycotoxins in a spiked feed sample.

### 4.6. Quantification of Mycotoxins and Statistical Analysis

The percentage adsorption of mycotoxins by each product was calculated as follows:

Adsorption (%) = (C_b_ − C_t_)/C_t_ × 100.

where C_b_ is the mycotoxin concentration in blank spiked buffer solutions (ng/mL); and C_t_, the amount of mycotoxin in the supernatant of sample (ng/mL). The data obtained were analysed using TargetLynx processing software (Waters, Wilmslow, UK) and Prism^®^ version 8 (San Diego, CA, USA). The means of treatments showing significant differences in two and three-way ANOVA were compared using Tukey’s honestly significance difference multiple-comparisons post-test. All statements of significance are based on the 0.05 level of probability.

## Figures and Tables

**Table 1:** Percentage adsorption of deoxynivalenol (DON), zearalenone (ZEN), fumonisin B1 (FB1), ochratoxin A (OTA), T-2 toxin and aflatoxin B1 (AFB1) by ten commercial feed additives in pH 3 and pH 7 buffer solutions *.

	Adsorbed Mycotoxin (%) (mean ± SD) **												
Category	Product	DON		ZEN		FB1		OTA		T-2		AFB1	
		pH 3	pH 7	pH 3	pH 7	pH 3	pH 7	pH 3	pH 7	pH 3	pH 7	pH 3	pH 7
Inorganic Additives	1	58 ± 0.5 ^b^	50 ± 2.9 ^b^	52 ± 0.9 ^b^	49 ± 1.1 ^a,b^	38 ± 1.2 ^b,c^	47 ± 0.9 ^a^	40 ± 1.6 ^b,c^	37 ± 1.1 ^b^	38 ± 0.7 ^b^	29 ± 1.1 ^c^	68 ± 0.8 ^a^	68 ± 1.6 ^a^
	2	33 ± 2.5 ^d^	31 ± 0.7 ^c^	32 ± 0.4 ^d^	31 ± 1.4 ^c^	33 ± 2.5 ^d^	30 ± 0.8 ^c^	12 ± 2.1 ^d^	12 ± 1.4 ^d^	08 ± 1.2 ^d^	06 ± 1.1 ^d^	49 ± 1.9 ^b^	41 ± 3.2 ^d^
	3	53 ± 1.3 ^c^	53 ± 1.8 ^b^	44 ± 1.2 ^c^	39 ± 2.3 ^c^	32 ± 3.1 ^d^	28 ± 0.6 ^c^	41 ± 1.4 ^b,c^	28 ± 1.6 ^c^	22 ± 1.5 ^c^	10 ± 1.2 ^d^	61 ± 3.4 ^a^	52 ± 2.7 ^c^
	4	29 ± 1.2 ^d^	22 ± 1.2 ^d^	27 ± 2.3 ^d^	20 ± 0.7 ^d^	29 ± 1.4 ^d^	22 ± 1.4 ^d^	05 ± 0.3 ^e^	02 ± 0.7 ^e^	09 ± 0.9 ^d^	09 ± 1.6 ^d^	51 ± 3.1 ^b^	47 ± 1.9 ^c,d^
Organic Additives	5	56 ± 1.9 ^c^	54 ± 1.5 ^b^	36 ± 1.4 ^d^	29 ± 0.9 ^c^	45 ± 0.9 ^b^	40 ± 1.9 ^b^	38 ± 0.8 ^b^	25 ± 2.3 ^c^	40 ± 2.2 ^b^	31 ± 1.1 ^c^	53 ± 4.2 ^b^	53 ± 2.1 ^c^
	6	55 ± 4.7 ^c^	55 ± 0.5 ^b^	56 ± 2.2 ^a^	56 ± 1.5 ^a^	51 ± 2.9 ^a^	50 ± 1.6 ^a^	60 ± 1.4 ^a^	56 ± 0.8 ^a^	55 ± 0.7 ^a^	56 ± 1.3 ^a^	65 ± 2.2 ^a^	65 ± 1.9 ^a^
	7	36 ± 3.2 ^c^	38 ± 1.1 ^c^	28 ± 0.3 ^d^	19 ± 1.2 ^d^	19 ± 1.7 ^e^	18 ± 2.2 ^d^	35 ± 2.5 ^c^	28 ± 1.2 ^c^	10 ± 1.3 ^d^	09 ± 1.3 ^d^	50 ± 1.9 ^b^	55 ± 1.6 ^c^
Mixed Additives	8	52 ± 1.2 ^c^	50 ± 0.6 ^b^	46 ± 1.9 ^c^	40 ± 1.6 ^b,c^	39 ± 2.3 ^b^	31 ± 0.7 ^c^	20 ± 2.2 ^d^	20 ± 1.3 ^c,d^	43 ± 1.6 ^b^	40 ± 2.1 ^b^	61 ± 2.3 ^a^	56 ± 1.9 ^c^
	9	72 ± 2.4 ^a^	71 ± 1.5 ^a^	55 ± 1.8 ^b^	52 ± 1.4 ^a,b^	42 ± 1.3 ^b^	25 ± 1.7 ^c^	49 ± 1.6 ^b^	37 ± 1.6 ^b^	55 ± 1.4 ^a^	53 ± 1.3 ^a^	63 ± 3.1 ^a^	60 ± 2.2 ^a,b^
	10	32 ± 1.6 ^d^	32 ± 1.4 ^c^	22 ± 1.2 ^e^	20 ± 1.1 ^d^	19 ± 1.9 ^e^	10 ± 1.7 ^e^	15 ± 1.4 ^d^	12 ± 0.9 ^d^	14 ± 1.6 ^d^	06 ± 1.9 ^d^	52 ± 2.5 ^b^	30 ± 1.6 ^e^

* Calculated in comparison to the control treatment with no feed additives. ** Values are means of three replicates. ^a–f^ Values labelled with the same superscript in a column are not significantly different (*p* > 0.05).

**Table 2:** Percentage adsorption of deoxynivalenol (DON), zearalenone (ZEN), fumonisin B1 (FB1), ochratoxin A (OTA), T-2 toxin and aflatoxin B1 (AFB1) by 10 commercially available feed additives in an in vitro model designed to mimic the gastrointestinal tract of a monogastric animal *.

Adsorbed Mycotoxin (%) (mean ± SD) **							
Category	Product	DON	ZEN	FB1	OTA	T-2	AFB1
Inorganic Additives	1	55 ± 3.1 ^b^	40 ± 2.2 ^b^	33 ± 3.6 ^c^	25 ± 2.5 ^c^	26 ± 1.3 ^c^	51 ± 2.9 ^b^
	2	39 ± 1.3 ^d^	29 ± 2.6 ^d^	20 ± 1.9 ^d^	18 ± 3.2 ^d^	04 ± 1.4 ^d^	53 ± 2.1 ^b^
	3	41 ± 1.6 ^d^	12 ± 1.2 ^f^	21 ± 2.3 ^d^	00	00	38 ± 1.5 ^c^
	4	31 ± 1.7 ^e^	18 ± 2.2 ^e^	20 ± 3.1 ^d^	00	02 ± 0.4 ^d^	42 ± 1.2 ^c^
Organic Additives	5	47 ± 1.9 ^c^	40 ± 2.4 ^b^	45 ± 2.1 ^b^	29 ± 1.5 ^b^	28 ± 1.3 ^c^	54 ± 2.2 ^b^
	6	55 ± 1.6 ^b^	53 ± 1.1 ^a^	51 ± 1.5 ^a^	52 ± 2.3 ^a^	56 ± 1.4 ^a^	62 ± 0.9 ^a^
	7	36 ± 2.2 ^e^	41 ± 2.5 ^b^	19 ± 0.6 ^d^	26 ± 0.9 ^c^	00	39 ± 1.4 ^c^
Mixed Additives	8	41 ± 3.3 ^d^	36 ± 1.7 ^c^	23 ± 1.4 ^d^	10 ± 2.1 ^e^	28 ± 1.6 ^c^	48 ± 1.9 ^b,c^
	9	61 ± 2.4 ^a^	53 ± 1.4 ^a^	35 ± 2.6 ^c^	32 ± 1.2 ^b^	35 ± 0.7 ^b^	58 ± 3.2 ^a^
	10	22 ± 1.8 ^f^	08 ±1.9 ^f^	00	00	00	29 ± 0.8 ^d^

* Calculated in comparison to the control treatment with no feed additives. ** Values are means of three replicates. ^a–f^ Values labelled with the same superscript in a column are not significantly different (*p* > 0.05).

**Table 3:** Composition and mode of actions (as stated on the product labels and manufacturers’ websites) of commercial feed additives claiming multiple-mycotoxin binding.

Category	Product	Main Composition	Mode of Action
Inorganic adsorbent	1	Modified aluminosilicates	Adsorption
	2	Bentonite	***
	3	Activated clay	Adsorption and Inactivation
	4	Montmorillonite	Adsorption
Organic adsorbent	5	Glucomannan	Adsorption and complexation
	6	Modified yeast cell wall	Adsorption
	7	Esterified glucomannan	***
Mixed adsorbent	8	Mixed silicates and yeast cell wall	***
	9	Aluminosilicate and enzyme	Adsorption and biotransformation
	10	Natural minerals and algae	Adsorption and degradation

*** no information provided.

**Table 4:** Signal suppression-enhancement/relative standard deviation (SSE/RSD) and limit of quantification (LOQ) obtained for deoxynivalenol (DON), zearalenone (ZEN), fumonisins B1 (FB1), ochratoxin A (OTA), T-2 and aflatoxin B1 (AFB1) and validated matrices—pH 3, pH 7 and gastrointestinal fluid (GF).

Matrix		DON	ZEN	FB1	OTA	T-2	AFB1
pH 3	SSE/RSD (%)	95/2.1	98/2.4	113/3.2	97/3.9	78/2.7	96/0.9
	LOQ (ng/mL)	2.5	2.5	5	0.4	2.5	0.13
pH 7	SSE/RSD (%)	78/2.6	97/2.7	83/3.1	102/4.1	88/3.4	100/1.2
	LOQ (ng/mL)	2.5	2.5	5	0.4	2.5	0.13
GF	SSE/RSD (%)	89/3.2	92/6.8	123/5.7	103/4.9	79/7.4	87/4.6
	LOQ (ng/mL)	2.5	5	5	0.8	5	0.25
